# The Relation of Vernier Acuity to Variations in Intensity and Wavelength

**DOI:** 10.3390/vision10040073

**Published:** 2026-09-28

**Authors:** Yaw Buabeng, Billy R. Hammond

**Affiliations:** Vision Sciences Laboratory, Behavioral and Brain Sciences Program, University of Georgia, Athens, GA 30602, USA; yaw.buabeng@uga.edu

**Keywords:** vernier acuity, action spectrum, fine spatial resolution, hyperacuity, wavelength

## Abstract

Clinical relevance: Vernier acuity (VA) is a common clinical metric used to evaluate cortical visual processing and macular health that is relatively independent of typical optical limitations (such as blurring). Background: Past studies have shown that vernier thresholds do not tend to vary according to blur, intensity and spectral content. This last variable, however, is the most inconsistent and has never been measured using relatively narrowband wavelengths. Methods: This study measured vernier thresholds (VT) as a function of wavelength (*n* = 15) and intensity (*n* = 5) in healthy young adults (mean age = 24.8 ± 2.3 years) with normal (or near-normal), corrected vision. VTs were tested using backlit apertures. Narrowband stimuli (420–660 nm) and broadband intensities were produced by a 1000 W Xenon arc lamp with interference filters. VTs, defined as the minimum detectable misalignments between two thin rectangular apertures, were recorded using a digital micrometer. Results: VTs did not vary significantly with wavelength (F (6,84) = 1.56, *p* = 0.17). Similarly, VTs did not vary significantly with the different broadband intensities (F (5,20) = 1.30, *p* =0.31). Conclusions: The stability of vernier acuity across these conditions indicates that hyperacuity performance is largely invariant to spectral composition and relatively stable across the limited photopic-to-mesopic intensity range that we tested. These findings are consistent with models proposing that vernier acuity depends primarily on post-receptoral neural mechanisms rather than optical or wavelength-dependent retinal factors.

## 1. Introduction

Vernier acuity (VA) is a form of acuity that reflects an individual’s ability to detect minute spatial misalignments between two visual elements [1,2,3,4]. Classic work by Westheimer [5] and others has demonstrated that vernier performance reflects post-receptoral spatial pooling and positional coding mechanisms rather than direct photoreceptor sampling, consistent with a cortical locus being the primary driver of hyperacuity judgments. Although it is not commonly used in clinical practice, studies have shown that VA is sensitive to visual dysfunction and can distinguish individuals with conditions as diverse as glaucoma, cataract, age-related macular degeneration and amblyopia [6,7,8,9].

The effects of luminance, contrast and temporal integration on vernier thresholds (VT) have been well documented [10]. For instance, Baker [11] showed vernier acuity rises with increasing illumination up to a certain threshold. A similar effect is seen in contrast [12]. VA varies with exposure times with longer exposures resulting in better performance than shorter ones due to temporal summation [13]. Narrow target separation and longer line lengths also improve VA [14]. VA is known to decrease with retinal eccentricity [15]. In contrast, the role of wavelength has received relatively little systematic attention. Wavelength could, in principle, influence spatial precision through several mechanisms, including chromatic aberration [16], wavelength-dependent differences in cone sampling density, or unequal engagement of chromatic and achromatic visual pathways. At lower photopic light levels, short-wavelength stimuli may also differentially engage rod photoreceptors, potentially influencing fine spatial judgments through rod–cone interactions. Studies that have explored these factors, however, have produced inconsistent results [16,17,18]. For example, early studies by Baker [11] and Foley-Fisher [13] tested vernier acuity under different lighting conditions (red, yellow, white and blue). Their results were consistent with an effect of wavelength, although the wavelength of peak performance was not consistent (alternated between red and yellow for some participants). Conversely, Waugh & Levi [12] and Krauskopf & Forte [18] argued that chromatic variations have no effects on VA. They suggested that once luminance of the target is optimal for detection, vernier acuity thresholds are similar across different wavelengths. This dependence on luminance for fine spatial tasks is consistent with Morgan and Aiba [19], who reported that VTs were higher for equiluminant than for luminance defined stimuli (i.e., color/wavelength mostly did not matter).

Many of the differences seen across studies are likely due to how the stimuli were presented and to differences in luminance control, spectral bandwidth, and psychophysical measurement methods. For example, recent studies tend to present the vernier stimuli with computer displays (black lines on a colored background) which often provide poor control of the wavelength properties of the stimulus. In the present study, the stimuli were presented as aperture-defined (backlit) vernier targets. A custom-built optical system with micrometric control of spatial displacement was used (see Figure 1). We assessed vernier performance across wavelengths and at multiple broadband intensity levels to determine whether fine resolution depends primarily on photopic drive or varies systematically with spectral composition. The aim of the present study was not to identify novel neural mechanisms of vernier acuity but to determine whether wavelength-dependent optical factors measurably influence spatial alignment judgments under tightly controlled spectral and geometric conditions.

## 2. Method

### 2.1. Study Design and Population

Twenty healthy young adults (50% female, 50% male; mean age = 24.8 years, SD = 2.33) participated. Descriptive statistics are reported in Table 1. Ocular history was obtained via self-report. Inclusion criteria were age 18–30 years and uncorrected visual acuity better than 20/40 in each eye, measured using a Snellen acuity chart. Visual acuity was tested without habitual correction, and the dominant eye was identified prior to testing. To avoid confounding effects of optical corrections, no participant wore glasses or contact lenses during the experiment. Although uncorrected viewing may introduce small refractive blur, all participants met acuity criteria (>20/40), and this approach ensured that wavelength-dependent effects from corrective optics were eliminated. Exclusion criteria included ocular conditions that could directly interfere with optical quality or light scatter (e.g., corneal infection, stye, or other anterior segment abnormalities).

Participants were recruited from the undergraduate and graduate student population at the University of Georgia. The study protocol was approved by the University of Georgia Institutional Review Board (PROJECT00009383). Procedures followed Good Clinical Practice Guidelines and the Declaration of Helsinki.

### 2.2. Apparatus and Stimuli

A detailed schematic of the apparatus can be found in Figure 1. A 1000-Watt Xenon arc lamp (Newport Corporation, Irvine, CA, USA) served as the light source (Newport Corporation). Light was focused through a system of achromatic lenses, heat and diffusing filters and passed through a circular neutral-density (ND) wedge that provided linear attenuation of light energy. This ND wedge was used to equalize luminance across conditions prior to spectral filtering.

Narrowband interference filters (20 nm half-bandpass) produced peak wavelengths of 420, 460, 500, 540, 580, 620, and 660 nm (Edmund Optics, Barrrington, NJ, USA). The relative energy of these sources is shown in Figure 2. A broadband white xenon condition was also included. Filtered light was projected onto an aperture-defined (2 × 2 inch bars, 0.05 inches wide) vernier stimulus (i.e., negative space). There was no surround field and participants were exposed to the stimulus as long as needed for them to make a judgement (this was usually within a few seconds). The upper arm of the cross could be displaced incrementally to the left or right relative to the central fixation point, allowing for precise control of horizontal misalignment during measurement (originally described in Hammond et al., 2020) [20]. At a viewing distance of 5.5 ft (1.68 m), the 0.05-inch aperture width subtended approximately 2.6 arcmin of visual angle. The overall bar length subtended approximately 1°44′. These geometric parameters are known to influence vernier thresholds 2. A collapsible baffle was positioned to block stray light between the moving and stationary portions of the cross during displacement, ensuring that only the intended stimulus elements were visible to the observer. A built-in digital micrometer recorded the displacements of the lines, with a minimum displacement of 0.03 mm, corresponding to approximately 3.7 arcsec at the 66-inch viewing distance. Extensive baffling throughout the optical system minimized stray light.

An adjustable chin-and-forehead rest positioned the participant 66 inches (≈5.5 ft) from the aperture shield, ensuring alignment with the optical axis.

### 2.3. Calibration and Wavelength Variation

Total photometric output was calibrated for each tested wavelength prior to testing. To ensure equal luminance across conditions, diffusing filters were inserted into the optical path for the 500 nm, 540 nm, and 580 nm wavelengths. A neutral-density wedge was then adjusted until illuminance was equalized at the aperture, as verified by a UDT Model S370 photodetector (4gte.com). 420 nm produced the least amount of luminance in this system; hence, in order to make all the wavelengths equal luminance, all were set to match this intensity. This luminance level, while low, was well above detection threshold for all participants and remained within the photopic to mesopic range for the stimulus size and viewing conditions used.

### 2.4. Intensity Variation

To determine the effect of different light intensities on VTs, a broadband condition was used across six distinct intensity groups on a subgroup of participants (*n* = 5) and using only the broadband white condition. The intensity was controlled by adjusting the ND wedge, in roughly equal logarithmic steps. These wedge settings corresponded to illuminance (lux) levels (measured at the plane of the stimulus) of: 39.14; 31.59; 24.64; 17.7; 10.7 and 3.39. Illuminance at the aperture was measured directly, with the UDT detector positioned directly abutting the aperture. We did not measure illuminance at the plane of the eye because the intensity was too low and the stimulus too small for the photo-detector to provide reliable measurements. The brightest intensity level was determined by setting the ND wedge to an output that was perceived as very bright yet still relatively comfortable for the subject.

### 2.5. Procedure

Testing was conducted in a darkened room (participants were not dark-adapted). Participants were instructed using illustrations of the cross-stimulus movement and testing was conducted under monocular viewing while the nondominant eye was covered with a patch. They rested their chin and forehead on a stabilized mount to minimize head motion. To ensure proper alignment, a small fixation post with a small hole was placed along the optical axis (in the exact position of the test stimulus 5.5 feet away); the ability to see the stimulus through that hole confirmed that the eye was in the correct position (this alignment rod was removed before testing).

Each trial began with the cross in its neutral position (upper arm precisely aligned along the vertical axis). The upper arm of the cross was then displaced slowly to the left or right until the subject reported perceiving a noticeable deviation from perfect alignment. This method of limits was chosen to allow fine control of displacement using the micrometer-driven optical system. Although the method of limits (widely used in classical vernier studies [5,21]) may introduce response bias and hysteresis effects, the randomized presentation and repeated measures design mitigated systematic bias across conditions.

The micrometer, built into the housing, was then used to measure the deviation. The cross was then reset to perfect physical alignment (zeroed) before the next trial. Each wavelength condition was tested three times in randomized order. Similarly, the six intensity groups in the broadband condition were tested three times each; trial order was randomized for each participant. Each experimental session lasted approximately an hour.

### 2.6. Statistical Analysis

All statistical analyses were performed using R version 4.3.2 within the RStudio integrated development environment (version 2023.12.0+369) and Microsoft Excel. One-way repeated-measures ANOVAs were conducted to assess the effects of wavelength on vernier performance and, separately, the effects of broadband light intensity on vernier performance. Bayesian repeated-measures ANOVAs were also conducted for both analyses to quantify the evidence for or against the null hypothesis of no effect of wavelength or intensity on vernier thresholds. For the Bayesian analyses, participant was specified as a random factor, and Bayes factors were calculated using the JZS prior. Statistical significance for the frequentist analyses was set at *p* < 0.05.

## 3. Results

Descriptive statistics for vernier thresholds across wavelength and illuminance conditions are summarized in Table 2 and Table 3. Wavelength had no statistically significant effect on vernier task performance across the seven tested wavelengths (420, 460, 500, 540, 580, 620, and 660 nm), *F*(6, 84) = 1.56, *p* = 0.169, partial η^2^ = 0.100. A Bayesian repeated-measures ANOVA provided additional support for the absence of a wavelength effect (*BF*_01_ = 3.12), indicating that the data were approximately 3.1 times more likely under the model without a wavelength effect than under the model including wavelength. This figure also shows the absolute energy of each waveband used for this determination. These energy levels reflect the adjustment of each waveband to achieve the same illuminance level.

For a small (*n* = 5) subset of participants, we also tested the effects of varying intensity on VT for the white-broadband condition only. The results indicated that illuminance had no statistically significant effect on vernier task performance, F(5, 20) = 1.30, *p* = 0.305, partial η^2^ = 0.245. This is shown in Figure 3.A Bayesian repeated-measures ANOVA provided additional support for the absence of an intensity effect (*BF*_01_ = 2.17), indicating that the data were approximately 2.2 times more likely under the model without an intensity effect than under the model including intensity. Thus, there were no significant differences in performance across the six illuminance levels (39.14, 31.59, 24.64, 17.70, 10.70, and 3.39 lux, measured at the aperture).

To examine the source of variability, within-observer variance across repeated wavelength measurements was compared with between-observer variance. Mean within-subject variance across wavelength conditions was 0.033 arcmin^2^ (SD ≈ 0.18 arcmin), whereas between-subject variance was 0.093 arcmin^2^ (SD ≈ 0.30 arcmin). Thus, between-subject variability was approximately 2.8 times greater than within-subject variability. This pattern indicates that the observed spread in thresholds primarily reflects stable individual differences in perceptual sensitivity or response criterion rather than trial-to-trial measurement noise.

## 4. Discussion

Across the seven wavebands (420–660 nm) and six intensity levels that were tested, VTs remained around 1 arcminute of visual angle. These findings are consistent with hyperacuity stimuli in that, under the specific stimulus, illuminance, and observer conditions tested here, the results are largely invariant with respect to spectral composition and relatively stable across modest photopic illuminance variations.

Although vernier acuity can reach hyperacuity levels (thresholds below 10 arcsec under optimized laboratory conditions), such performance depends strongly on stimulus geometry, contrast, and psychophysical procedure. The present study employed aperture-defined bars approximately 2.6 arcmin wide, presented at moderate photopic to mesopic illuminance and measured using a method-of-limits procedure. Thresholds were measured using an ascending series from alignment to detectable misalignment. Because descending series were not tested, potential direction-dependent or response-criterion effects cannot be excluded. Future studies could compare ascending and descending series to determine whether adjustment direction influences vernier thresholds. Both stimulus width and adjustment-based methods are known to elevate thresholds relative to forced-choice paradigms using thin, high-contrast lines. The thresholds observed here therefore reflect functional alignment acuity under tightly controlled optical conditions rather than maximal hyperacuity limits (more similar to Engles et al., 2007 [22]).

Baker [11] (using transparency slides and projectors) and Foley-Fisher [13] (using an optical system with movable lines on two planes of glass in a collimated light path) found that VTs were better under yellow and red-light conditions compared to blue. Later studies [19,23] found that, if luminance is controlled, wavelength effects disappear. Many past studies, however, used broad spectral sources or filtered projectors with incomplete luminance control. Computer-based studies (the current norm) rely on phosphor or LED-based emissions with comparatively poor control of wavelength and intensity. Under these exacting conditions, wavelength did not alter vernier performance, even at short wavelengths (420–460 nm), where optical issues like longitudinal chromatic aberration should be maximal and differences in the spatial sampling density and relative distribution of L-, M-, and S-cones across the photoreceptor mosaic would likely play a role. Although short-wavelength stimuli may also increase the likelihood of rod participation at lower photopic levels, the absence of systematic wavelength effects suggests that any rod contribution under these conditions did not measurably influence vernier performance [23]. This supports the hypothesis that vernier judgments depend, not on early ocular or retinal determinants, but rather on later, more centrally determined spatial mechanisms [2,3,14].

Previous work has also been inconsistent regarding the effects of luminance changes. Baker [11] and Beard et al. [10], for instance, found that VTs improve with luminance until a plateau is reached. In this study, we found that changes in luminance (using broadband light and a smaller subset of our sample, *n* = 5) did not change VTs. The broadband intensity manipulation covered a relatively restricted range (3.39–39.14 lux measured at the plane of the stimulus), and the small stimulus produced illuminance at the eye that was below the reliable measurement range of our photodetector.

Participants were young, visually normal adults with relatively clear ocular media. Results may differ in older individuals or in populations with increased short-wavelength scatter, lens yellowing, or intraocular lenses. The sample was predominantly White (85%) and entirely non-Hispanic, limiting our ability to detect effects associated with broader variation in ocular pigmentation. We also did not measure macular pigment optical density, which limited our ability to assess whether individual differences in short-wavelength filtering influenced thresholds, although Engles et al. (2007) found little evidence of such an association [22]. Only horizontal offsets were tested, and vernier performance may differ for oblique orientations due to the oblique effect [24]. The luminance manipulation was conducted in a small subset of participants and across a restricted luminance range, limiting generalization to broader luminance conditions.

In conclusion, we found no evidence that wavelength measurably affected hyperacuity performance for aperture-defined vernier stimuli in young, visually normal observers. This is likely why spectral filters (like macular pigment or tinted contact lenses) do not tend to preferentially improve vernier acuity by filtering out highly aberrated short-wave light [22,25].

The findings provide one of the few tests of vernier acuity using narrowband interference-filter stimuli under tightly controlled optical geometry. Future work should determine wavelength-specific illuminance thresholds at which vernier performance begins to degrade. Such data may help dissociate photoreceptor-level from post-receptoral contributions to spatial alignment judgments.

## Figures and Tables

**Figure 1:**
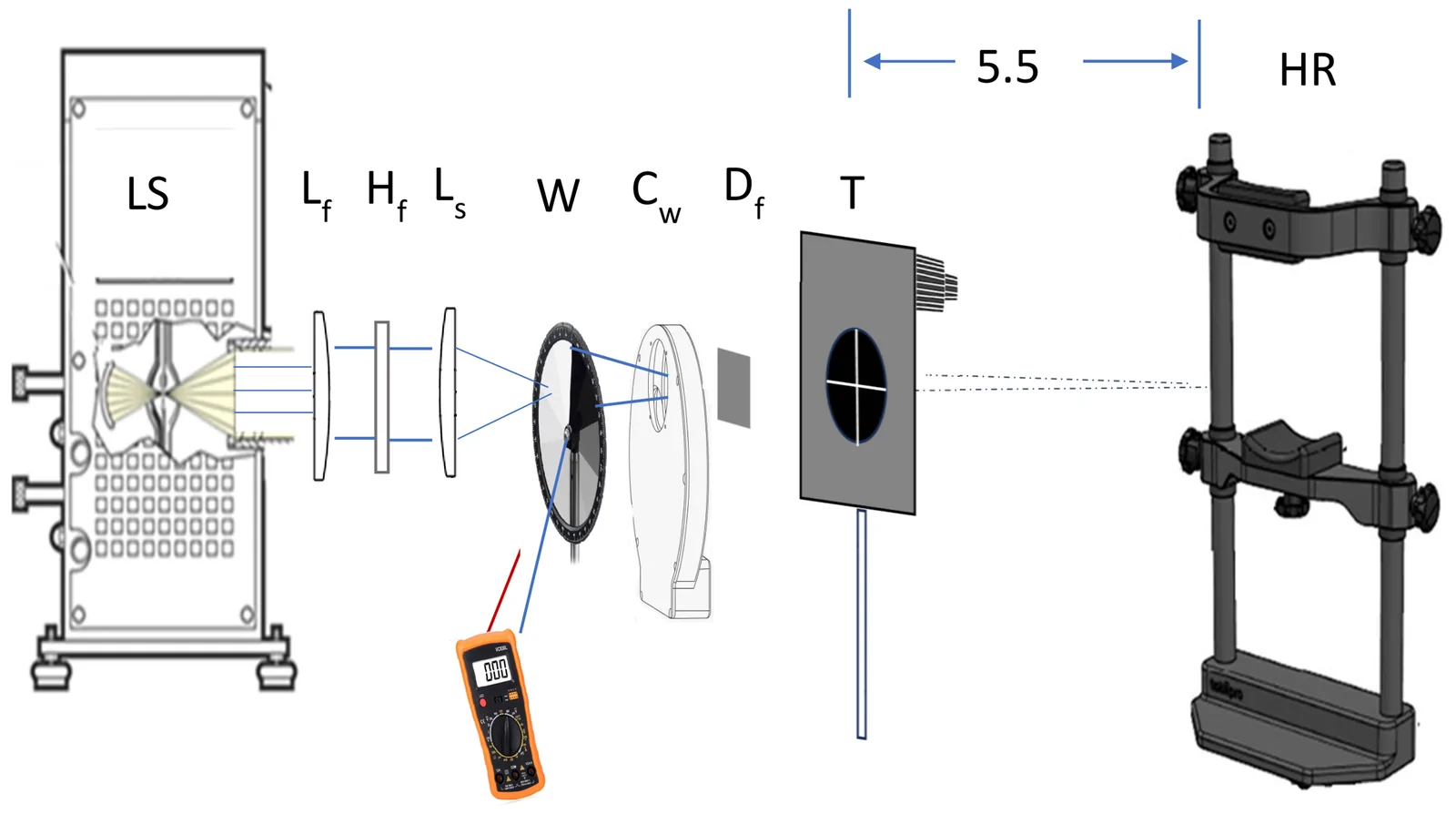
A conceptual schematic of the device used to measure vernier acuity across wavelengths and varying light intensities. LS = Light source; HR = Headrest; T = Target; W = Wedge; L_f_ = First lens; H_f_ = Heat filter; L_s_ = Second lens; C_w_ = Circular Wheel; D_f_ = Diffuser.

**Figure 2:**
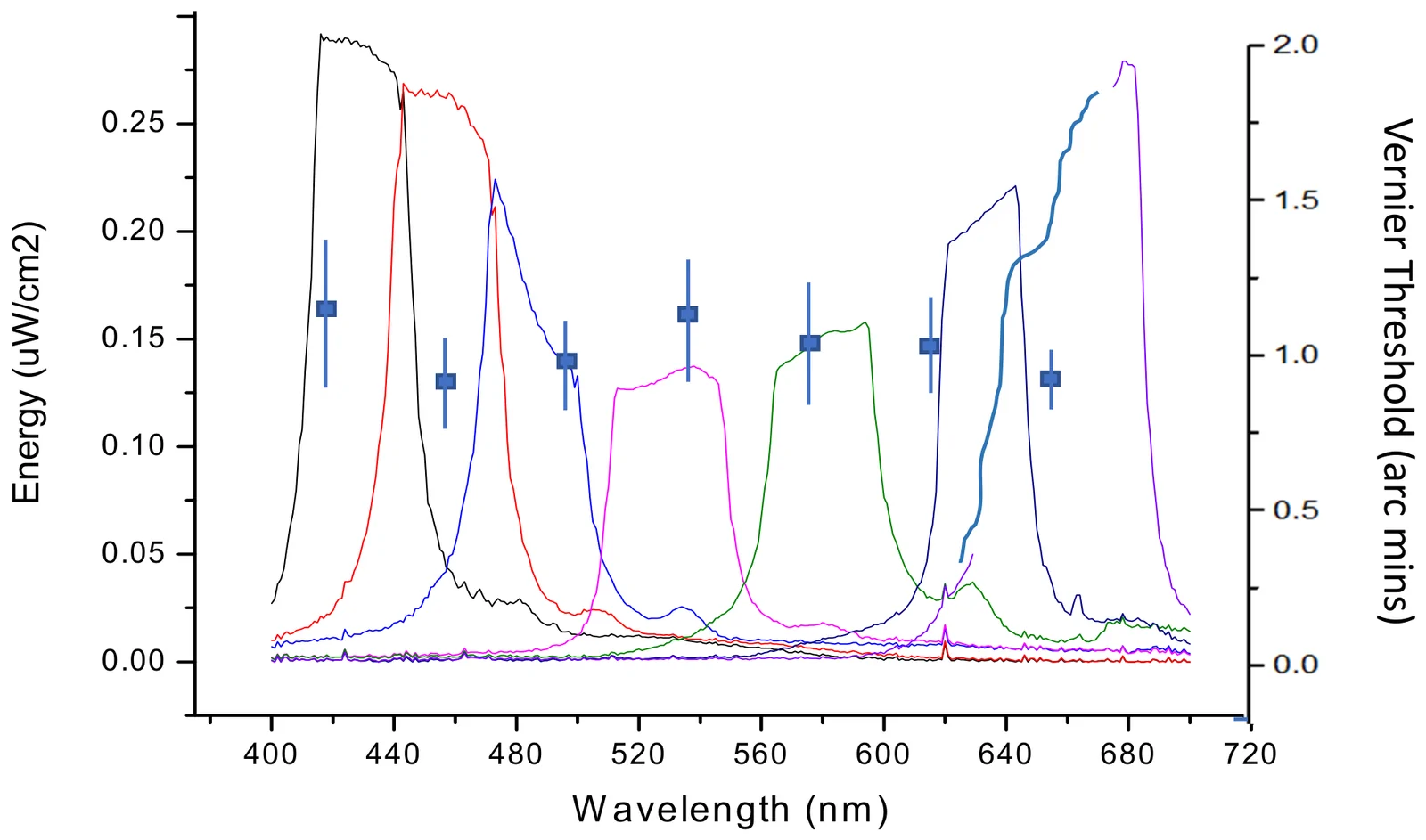
Mean vernier thresholds (±SEM) are plotted alongside normalized spectral power distributions of the narrowband interference filters. Thresholds were obtained under equalized illuminance conditions.

**Figure 3:**
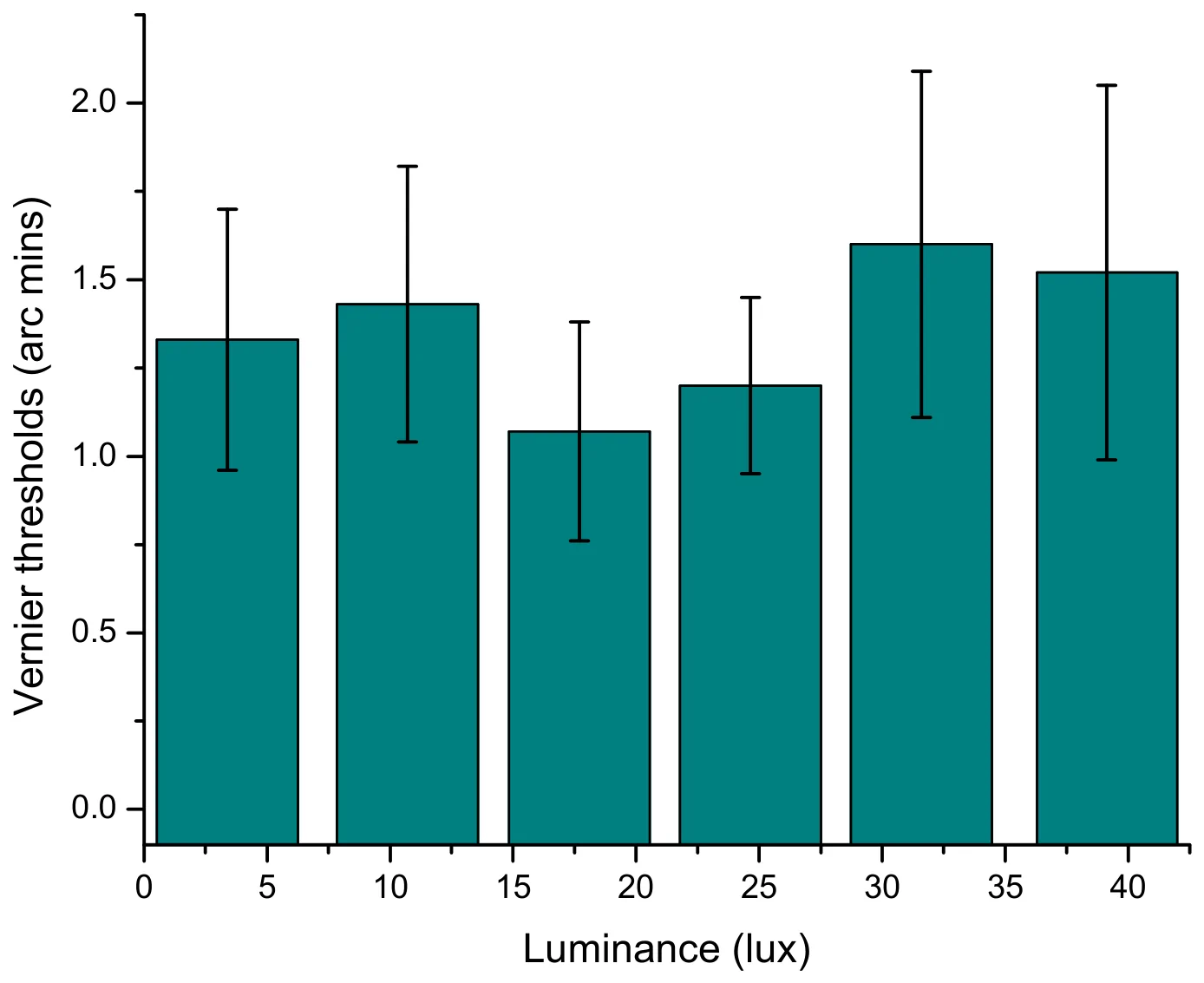
Vernier thresholds (mean ± SD) across illuminance levels for the broadband condition (*n* = 5). Standard deviation is shown rather than SEM due to the smaller sample size in this subset.

**Table 1:** Descriptive statistics (*n* = 20).

Variable	Average	SD	Range
**Age**	24.8	2.33	20 to 29
**Race**	15% Black/African American 85% White/Caucasian	N/A	N/A
**Gender**	50% male 50% female	N/A	N/A
**Ethnicity**	100% non-Hispanic	N/A	N/A

**Table 2:** Descriptive Statistics (*n* = 15) for wavelengths (values expressed in minutes of visual angle).

Wavelengths (nm)	Average	SD	Range
420	1.13	0.87	0.49–3.26
460	0.90	0.68	0.35–3.24
500	0.96	0.65	0.37–3.03
540	1.10	0.75	0.37–2.71
580	1.02	0.79	0.33–2.87
620	1.00	0.61	0.16–2.56
660	0.71	0.36	0.27–1.60

**Table 3:** Descriptive Statistics (*n* = 5) for illuminance (values expressed in minutes of visual angle).

Illuminance (Lux) at the Plane of the Stimulus	Average	SD	Range
3.4	1.33	0.81	0.66–2.73
10.7	1.43	0.87	0.82–2.83
17.7	1.07	0.67	0.49–2.15
24.64	1.20	0.53	0.70–2.03
31.59	1.60	1.09	0.68–3.10
39.14	1.52	1.20	0.59–3.36

## Data Availability

The original contributions presented in this study are included in the article Further inquiries can be directed to the corresponding author.
